# Reviewer acknowledgement 2015

**DOI:** 10.1186/s13023-015-0245-6

**Published:** 2015-03-28

**Authors:** Ségolène Aymé

**Affiliations:** Orphanet - INSERM US14, Rare Disease Platform, 96 rue Didot, 75014 Paris, France

## Abstract

The Editors of *Orphanet Journal of Rare Diseases* would like to thank all our reviewers who have contributed to the journal in volume 9 (2014).

**Lauri Aaltonen**

Finland

**Damiano Abeni**

Italy

**Mario Abinun**

United Kingdom

**Marc Abramowicz**

Belgium

**Maria Isabel Achatz**

Brazil

**Maria Acosta**

United States of America

**David Adams**

France

**Johannes Aerts**

Netherlands

**Adnan Al-Araji**

United Kingdom

**Laurent Alberti**

France

**Fowzan Alkuraya**

Saudi Arabia

**Hamoud Al-Mousa**

Saudi Arabia

**Maud Els-Marie Andersson**

Norway

**Hans Andersson**

United States of America

**Corrado Angelini**

Italy

**Lieven Annemans**

Belgium

**Simone Ardern-Holmes**

Australia

**Zohar Argov**

Israel

**Peter Arkwright**

United Kingdom

**Thaís Armangue**

Spain

**Andrea Artoni**

Italy

**David Atlan**

Switzerland

**Shahram Attarian**

France

**Laura Audi**

Spain

**Isabelle Audo**

France

**Karen Avraham**

Israel

**Adrianus Bakermans**

Netherlands

**Lilia Baldazzi**

Italy

**Irina Bancos**

United States of America

**Ayla Barutchu**

Austria

**Murat Bastepe**

United States of America

**Jean Bastin**

France

**Neil Basu**

United Kingdom

**Genevieve Baujat**

France

**Matthias Baumgartner**

Switzerland

**Omer Faruk Bayrak**

Turkey

**Tommaso Beccari**

Italy

**Michael Beck**

Germany

**Karin Becke**

Germany

**Bjoern Beermann**

Sweden

**Nadia Belmatug**

France

**Bruno Bembi**

Italy

**Raida Ben Salah**

Tunisia

**Ilan Ben-Zvi**

Israel

**José Berciano**

Spain

**Carsten Bergmann**

Germany

**Peter Berlit**

Germany

**Laura Bernardini**

Italy

**Michael Berndt**

Australia

**Erik Berntorp**

Sweden

**Christophe Beroud**

France

**Enrico Bertini**

Italy

**Hunter Best**

United States of America

**Conceicao Bettencourt**

United Kingdom

**Jorge Bevilacqua**

Chile

**Marino Bianchin**

Brazil

**Marieke Biegstraaten**

Netherlands

**Maria Bitner-Glindzicz**

United Kingdom

**Douglas Bittel**

United States of America

**Craig Blackstone**

United States of America

**Carl Blankart**

Germany

**Clemens Bloetzer**

Switzerland

**Olivia Boccara**

France

**Olaf Bodamer**

United States of America

**Sylvia Boesch**

Austria

**Amanda Bok**

Belgium

**Luisa Bonafé**

Switzerland

**Vincenzo Bonifati**

Netherlands

**Josh Bonkowsky**

United States of America

**Mieke Boon**

Belgium

**David Booth**

Australia

**Luca Borradori**

Switzerland

**Annet Bosch**

Netherlands

**Laurence Bouillet**

France

**Rose-Mary Boustany**

Lebanon

**Breidge Boyle**

United Kingdom

**Helen Boyle**

France

**Adrian Brady**

Ireland

**Roscoe Brady**

United States of America

**Darrin Brager**

United States of America

**Mariana Brait**

United States of America

**Allison Brashear**

United States of America

**Nancy Braverman**

Canada

**Guilherme Bresciani**

Chile

**Corstiaan Breugem**

Netherlands

**Michael Briggs**

United Kingdom

**Steven Brody**

United States of America

**Alexander Broomfield**

United Kingdom

**Andrew Brown**

Australia

**Steve Brown**

United Kingdom

**Michael Brudno**

Canada

**Carlo Brugnara**

United States of America

**Cecilia Bucci**

Italy

**Peter Burgard**

Germany

**Lydie Burglen**

France

**Alberto Burlina**

Italy

**Patricia Burrows**

United States of America

**Monica Busse**

United Kingdom

**Kimberly Bussey**

United States of America

**Peter Byers**

United States of America

**Teresa Caballero**

Spain

**Philippe Camus**

France

**Andrew Cant**

United Kingdom

**Luca Cantarini**

Italy

**Valerio Carelli**

Italy

**Rachel Carling**

United Kingdom

**Carlos Casasnovas**

Spain

**Philippe Cassier**

France

**Marco Castori**

Italy

**Spero Cataland**

United States of America

**Salvatore Cazzato**

Italy

**Irene Ceballos-Picot**

France

**Stephen Cederbaum**

United States of America

**Marie-Camille Chaumais**

France

**David Cheillan**

France

**Jamel Chelly**

France

**Jose Chies**

Brazil

**Patrick Chinnery**

United Kingdom

**Carlo Chizzolini**

Switzerland

**Bodemer Christine**

France

**Brian Hon-yin Chung**

Hong Kong

**Marco Cicardi**

Italy

**Joe Clarke**

Canada

**Lorne Clarke**

Canada

**Monique Cloherty**

United Kingdom

**Harold Collard**

United States of America

**Lucy Collis**

United Kingdom

**Isabel Conceição**

Portugal

**Robert Cooper**

United Kingdom

**Valerie Cormier-Daire**

France

**Harriet Corvol**

France

**Vincent Cottin**

France

**Timothy Martin Cox**

United Kingdom

**Daniela Curti**

Italy

**Bogdan Cylwik**

Poland

**Bruno Dallapiccola**

Italy

**Anne D'Andon**

France

**Orbach Daniel**

France

**Fereydoun Davatchi**

Iran

**Hugh Dawkins**

Australia

**Julie De Backer**

Belgium

**Thomas de Broucker**

France

**Arjan de Brouwer**

Netherlands

**Jessica de Greef**

United States of America

**Gerard de Jong**

Australia

**Gérard de Pouvourville**

France

**Oriol de Solà-Morales**

Spain

**Hubert de Verneuil**

France

**Maria Debiec-Rychter**

Bhutan

**Patrick Deegan**

United Kingdom

**Bertrand Degos**

France

**Christine Delprat**

France

**Federica Deodato**

Italy

**David Derry**

United Kingdom

**Vincent Des Portes**

France

**Rachel Desailloud**

France

**Karl Desch**

United States of America

**Robert Desnick**

United States of America

**Grazia Devigili**

Italy

**Ferdinand Dhombres**

France

**Nataliya Di Donato**

Germany

**Maria Cristina Digilio**

Italy

**Carlo Dionisi-Vici**

Italy

**Jean Donadieu**

France

**Maria Teresa Dotti**

Italy

**Diane Doummar**

France

**Sophie Dupuis-Girod**

France

**Marinus Duran**

Netherlands

**Andrew Dwyer**

Switzerland

**Melanie Ehrlich**

United States of America

**Christa Einspieler**

Austria

**Aziz El-Amraoui**

France

**Peter Elias**

United States of America

**Paul Engel**

Ireland

**Gregory Enns**

United States of America

**Robert Eppsteiner**

United States of America

**Kelly Evans**

United States of America

**Olivier Fain**

France

**Julien Faure**

France

**François Feillet**

France

**Jilles Fermont**

United Kingdom

**Sebastiano Filetti**

Italy

**Andrea Finocchi**

Italy

**Josef Finsterer**

Austria

**Gilberto Fischer**

Brazil

**Kathelijn Fischer**

Netherlands

**William Foulkes**

Canada

**Brunella Franco**

Italy

**Michael Frank**

United States of America

**Hudson Freeze**

United States of America

**Roseline Froissart**

France

**Tod Fullston**

Australia

**Vilma Gabbay**

United States of America

**Andreas Gal**

Germany

**Livia Garavelli**

Italy

**Gemma Gatta**

Italy

**Andrew Gennery**

United Kingdom

**Piero Giordano**

Netherlands

**Santhosh Girirajan**

United States of America

**Emma Glamuzina**

New Zealand

**Grainne Gorman**

United Kingdom

**Philippe Gorry**

France

**Gilles Grateau**

France

**Josep Grau**

Spain

**Paolo Gresele**

Italy

**Robert Griggs**

United States of America

**Daniel Grinberg**

Spain

**Stephanie Grunewald**

United Kingdom

**Ghiabe Guibinga**

United States of America

**Fiorella Gurrieri**

Italy

**Johannes Häberle**

Switzerland

**Eric Hachulla**

France

**Alice Hadchouel**

France

**Smail Hadj-Rabia**

France

**Xianlin Han**

United States of America

**Jennifer Hand**

United States of America

**Karen Hansen**

United States of America

**Sergio Harari**

Italy

**Jean-Pierre Hardelin**

France

**Paul Harmatz**

United States of America

**Vanessa Harrar**

United Kingdom

**Dominik Hartl**

Germany

**Cristina Has**

Germany

**Daiichiro Hasegawa**

Japan

**Karen Heath**

Spain

**Adam Heathfield**

United Kingdom

**Chad Heatwole**

United States of America

**Matthew Helbert**

United Kingdom

**Christian Hendriksz**

United Kingdom

**Elizabeth Hernberg-Stahl**

Sweden

**Frederik Hes**

Netherlands

**James Heubi**

United States of America

**Wendy Heywood**

United Kingdom

**Ralf-Dieter Hilgers**

Germany

**Virginie Hivert**

France

**Jeffrey Hoag**

United States of America

**Franziska Hoche**

United States of America

**Georg Hoffmann**

Germany

**Carla Hollak**

Netherlands

**Aidan Hollis**

Canada

**Elisabeth Holme**

Sweden

**Manfred Hönig**

Germany

**Sander Michel Houten**

United States of America

**Riekelt Houtkooper**

Netherlands

**Lucile Houyel**

France

**Charlotte Höybye**

Sweden

**Ieuan Hughes**

United Kingdom

**Wills Hughes-Wilson**

Sweden

**Peter Humphreys**

Canada

**Susan Huson**

United Kingdom

**Adam Hutchings**

United Kingdom

**Dai Inoue**

Japan

**Alfonso Iorio**

Canada

**Desguerre Isabelle**

France

**Kinya Ishikawa**

Japan

**Peter Itin**

Switzerland

**Kentaro Iwata**

Japan

**Marshall Jacobs**

United States of America

**Gritta Janka**

Germany

**Clara Jaramillo**

France

**Giridhara Rao Jayandharan**

India

**Frederic Jehan**

France

**Zhanjun Jia**

United States of America

**Hong Jiang**

United States of America

**Xuntian Jiang**

United States of America

**Adriano Jimenez**

Spain

**Dong-Kyu Jin**

South Korea

**Hyder Jinnah**

United States of America

**Micallef Joelle**

France

**Colin Johnson**

United Kingdom

**Pascal Joly**

France

**Elizabeth Jones**

United Kingdom

**Roberta Joppi**

Italy

**Albena Jordanova**

Belgium

**Bardakdjian-Michau Josiane**

France

**Harald Jueppner**

United States of America

**Daria Julkowska**

France

**Hyunmin Jung**

United States of America

**Marshall Kadin**

United States of America

**Harald Kaemmerer**

Germany

**Rim Kahloun**

Tunisia

**Noor Kalsheker**

United Kingdom

**Tim Kanters**

Netherlands

**Paige Kaplan**

United States of America

**Daniela Karall**

Austria

**Henrik Karring**

Denmark

**Antonis Kattamis**

Greece

**David Kelsell**

United Kingdom

**David Ketteridge**

Australia

**Babak Khoshnood**

France

**Joseph Khoury**

United States of America

**Jung-Wook Kim**

South Korea

**Dimitra Kiritsi**

Germany

**Peter Kisfali**

Hungary

**Philipp Klaritsch**

Austria

**Tjitske Kleefstra**

Netherlands

**Joerg Klepper**

Germany

**Armin Koch**

Germany

**Tomoko Kodama**

Japan

**Franz Koenig**

Austria

**Sebastian Köhler**

Germany

**Alfried Kohlschuetter**

Germany

**Cornelia Kornblum**

Germany

**Ina Kötter**

Germany

**Markus Kraemer**

Germany

**Wolfram Kress**

Germany

**Usha Krishnan**

Australia

**Lauren Krivitzky**

United States of America

**Roland Kuiper**

Netherlands

**Manju Kurian**

United Kingdom

**François Labarthe**

France

**Philippe Labrune**

France

**Robin Lachmann**

United Kingdom

**Pascal Laforêt**

France

**Martin Laimer**

Austria

**Nigel Laing**

Australia

**Marc Lalande**

United States of America

**Wayne Lam**

United Kingdom

**Michele Lambert**

United States of America

**Linn Landrø**

Norway

**Mirjam Langeveld**

Netherlands

**Craig Langman**

United States of America

**Pablo Lapunzina**

Spain

**Lidia Larizza**

Italy

**Alexandre Lautrette**

France

**Matthias Lavens**

Belgium

**Christine Lavery**

United Kingdom

**Conxi Lázaro**

Spain

**Romain Lazor**

Switzerland

**Francois Le Loarer**

France

**Alexandra Leary**

France

**Caroline Leclercq**

France

**Carolina Lemos**

Portugal

**Helen Leonard**

Australia

**Jana Lepiksone**

Latvia

**Li Lin**

United States of America

**Ales Linhart**

Czech Republic

**Gabor Egon Linthorst**

Netherlands

**Christian Lobsiger**

France

**Hilary Longhurst**

United Kingdom

**Nicola Longo**

United States of America

**Maurizio Luisetti**

Italy

**Rigmor Lundby**

Norway

**Svetlana Lutsenko**

United States of America

**Alan Ma**

Australia

**Lijiang Ma**

United States of America

**Anita MacDonald**

United Kingdom

**Karen Lindhardt Madsen**

Denmark

**Esther Maier**

Germany

**Michael Makris**

United Kingdom

**David Malkin**

Canada

**Anwar Suleman Mall**

South Africa

**Renzo Manara**

Italy

**Maria Elisa Mancuso**

Italy

**Hanna Mandel**

Israel

**Pier Mannuccio Mannucci**

Italy

**Renato Mantegazza**

Italy

**Mario Manto**

Belgium

**Pineda Marfa**

Spain

**Christophe Mariat**

France

**Sandrine Marie**

Belgium

**Branka Marinovic**

Croatia

**Simone Martinelli**

Italy

**Rudolf Martini**

Germany

**Esmeralda Martins**

Portugal

**Jose Mascaro**

Spain

**Mariana Maschietto**

Brazil

**Andrea Masotti**

Italy

**Yves Maury**

France

**Alison May**

United Kingdom

**Jim McGill**

Australia

**John McGrath**

United Kingdom

**Anil Mehta**

United Kingdom

**Meir Mei-Zahav**

Israel

**Daniela Melis**

Italy

**Eugen Mengel**

Germany

**Fred Menko**

Netherlands

**Björn Menten**

Belgium

**Saadet Mercimek-Mahmutoglu**

Canada

**Maria Merino**

United States of America

**Peter Merkus**

Netherlands

**Lawrence Merritt**

United States of America

**Fredrik Mertens**

Sweden

**Aine Merwick**

United Kingdom

**Ludwine Messiaen**

United States of America

**Alexander Miethke**

United States of America

**Jose Millan**

Spain

**Akio Mimori**

Japan

**Snezana Minic**

Serbia

**Pramod Mistry**

United States of America

**Aladdin Mohammad**

Sweden

**Leo Monnens**

Netherlands

**Adriana Montano**

United States of America

**Michael Montemurro**

Switzerland

**Ute Moog**

Germany

**Eva Morava**

United States of America

**Thomas Morel**

Belgium

**Takayuki Morisaki**

Japan

**Jean-François Mornex**

France

**Richard Moscicki**

United States of America

**Andrew Mumford**

United Kingdom

**Craig Munns**

Australia

**Francisco Muñoz-Beamud**

Spain

**Alessandro Mussa**

Italy

**Martha Nance**

United States of America

**Nadia Nathan**

France

**Katherine Needleman**

United States of America

**Juergen Neesen**

Austria

**John Neidhardt**

Germany

**Giovanni Neri**

Italy

**Bénédicte Neven**

France

**Elena Nicod**

United Kingdom

**Lawrence Nogee**

United States of America

**Patrice Nony**

France

**Declan Noone**

Ireland

**William Nyhan**

United States of America

**Anita Oberbauer**

United States of America

**Sarah O'Brien**

United States of America

**Simon Olpin**

United Kingdom

**Rikke Olsen**

Denmark

**Osamu Onodera**

Japan

**Antonio Orlacchio**

Italy

**Augusto Orlandi**

Italy

**Massimiliano Paganelli**

Canada

**Michael Paine**

United States of America

**Francesc Palau**

Spain

**Mario Palermo**

Italy

**Michael Palladino**

United States of America

**Massimo Pandolfo**

Belgium

**Helen Papadaki**

Greece

**Joseph Parambil**

United States of America

**Davide Pareyson**

Italy

**Rossella Parini**

Italy

**Samantha Parker**

France

**Eduard Paschke**

Austria

**Juan Pascual**

United States of America

**Gregory Pastores**

Ireland

**Millan Patel**

Canada

**Marc Patterson**

United States of America

**Juha Peltonen**

Finland

**Luiz Penalva**

United States of America

**Susan Perlman**

United States of America

**Isabelle Perrault**

France

**Luca Persani**

Italy

**Christine Petit**

France

**Flora Peyvandi**

Italy

**Eline Picavet**

Belgium

**Herwig Pieringer**

Austria

**Nicola Pimpinelli**

Italy

**Vinciane Pirard**

Belgium

**Amélie Piton**

France

**Violaine Plante Bordeneuve**

France

**Petr Pohunek**

Czech Republic

**Chris Porada**

United States of America

**Grzegorz Porebski**

Poland

**Siamak Pourhassan**

Germany

**Nicolai Preisler**

Denmark

**Gaetan Prevost**

France

**Ewa Pronicka**

Poland

**Hervé Puy**

France

**Lori Quinn**

United Kingdom

**Jes Rahbek**

Denmark

**Julian Raiman**

Canada

**Fabienne Rajas**

France

**Gopi Rangan**

Australia

**A. Koneti Rao**

United States of America

**David Rapoport**

United States of America

**Ana Maria Rath**

France

**Bernard Ravina**

United States of America

**Arnold Reuser**

Netherlands

**Nima Rezaei**

Iran

**Vincent M. Riccardi**

United States of America

**Stéphane Richard**

France

**Antoni Riera-Mestre**

Spain

**Eve Roberts**

Canada

**Richard Roberts**

United States of America

**Peter Robinson**

Germany

**Xavier Roca**

Singapore

**Diana Rodriguez**

France

**Charlotte Rodwell**

France

**Catherine Rose**

Australia

**Frits Rosendaal**

Netherlands

**Margalit Rosenkranz**

United States of America

**Thomas Rossor**

United Kingdom

**Vicente Rubio**

Spain

**Heiko Runz**

Germany

**David Saadoun**

France

**Denise Sabatino**

United States of America

**Carlo Sabbà**

Italy

**Carsten Saft**

Germany

**Jose-Alain Sahel**

France

**Agnes Saint Raymond**

United Kingdom

**Lynn Sakai**

United States of America

**Carlo Salvarani**

Italy

**Victoria San Antonio-Arce**

Germany

**Luca Sangiorgi**

Italy

**Damien Sanlaville**

France

**Gijs Santen**

Netherlands

**René Santer**

Germany

**Paramala Santosh**

United Kingdom

**Eliane Sardh**

Sweden

**Swati Sathe**

United States of America

**Nadia Sawicka-Gutaj**

Poland

**John Sayer**

United Kingdom

**Fernando Scaglia**

United States of America

**Angela Scheuerle**

United States of America

**Manuel Schiff**

France

**Raphael Schiffmann**

United States of America

**Nicolas Schleinitz**

France

**Enno Schmidt**

Germany

**Artur Schmidtchen**

Sweden

**Matthias Schmuth**

Austria

**Benedikt Schoser**

Germany

**Andreas Schulze**

Canada

**Ida Vanessa Schwartz**

Brazil

**Marie Scully**

United Kingdom

**Matthew Seaman**

United Kingdom

**Michael Segal**

United States of America

**Susana Seixas**

Portugal

**Duygu Selcen**

United States of America

**Robert Semple**

United Kingdom

**Bruno Sepodes**

Portugal

**Rony Sfeir**

France

**William Shafer**

United States of America

**Tooru Shimosegawa**

Japan

**Satoru Shinkuma**

Japan

**Yehuda Shoenfeld**

Israel

**Cheryl Shoubridge**

Australia

**Gabriele Siciliano**

Italy

**Eduardo Silva**

Portugal

**Helga Silva**

Brazil

**Steven Simoens**

Belgium

**Anna Simon**

Netherlands

**Alessandro Simonati**

Italy

**Natalie Sims**

Australia

**Rani Singh**

United States of America

**Nicolas Sireau**

United Kingdom

**Cassian Sitaru**

Germany

**Deborah Sival**

Netherlands

**Flemming Skovby**

Denmark

**Anne Slavotinek**

United States of America

**Jan Smeitink**

Netherlands

**Sarah Smithson**

United Kingdom

**Etienne Sochett**

Canada

**Javier Soto**

Spain

**Paolo Spagnolo**

Switzerland

**Carsten Speckmann**

Germany

**Ute Spiekerkoetter**

Germany

**Malte Spielmann**

Germany

**Isabel Spier**

Germany

**Edoardo Spina**

Italy

**Lewis Spitz**

United Kingdom

**Eli Sprecher**

Israel

**Doris Staab**

Germany

**Miriam Stampfer**

Germany

**Matthew Steensma**

United States of America

**Barry Stein**

United States of America

**Robert Steinfeld**

Germany

**Giovanni Stevanin**

France

**Fiona Stewart**

United Kingdom

**Constantine Stratakis**

United States of America

**Arnold Strauss**

United States of America

**Pasquale Striano**

Italy

**Shigeaki Suzuki**

Japan

**Matthis Synofzik**

Germany

**Agnes Szilagyi**

Hungary

**Ikumi Tamai**

Japan

**Ricardo Tapia**

Mexico

**Abdellatif Tazi**

France

**Wei-Peng Teo**

Australia

**Swee Lay Thein**

United Kingdom

**Jess Thoene**

United States of America

**Dagmar Timmann**

Germany

**Vincent Timmerman**

Belgium

**Brian Tom**

United Kingdom

**Roser Torra**

Spain

**Rosa J Torres**

Spain

**Alberto Tosetto**

Italy

**Isabelle Touitou**

France

**Mondher Toumi**

France

**Juan Tovar**

Spain

**Elias Traboulsi**

United States of America

**Lisbeth Tranebjaerg**

Denmark

**Bruce Trapnell**

United States of America

**Eileen Treacy**

Ireland

**Edward Tuddenham**

United Kingdom

**Anna Tylki-Szymanska**

Poland

**Shin-ichi Usami**

Japan

**Graziella Uziel**

Italy

**Antra Valdmane**

Latvia

**Enza Maria Valente**

Italy

**Dominique Valla**

French Polynesia

**G. Caroline Van Baal**

Netherlands

**H. Marijke van den Berg**

Netherlands

**Silvere van der Maarel**

Netherlands

**Ans van der Ploeg**

Netherlands

**Ingeborg van der Tweel**

Netherlands

**Keith Van Haren**

United States of America

**Veronica van Heyningen**

United Kingdom

**Veronica van Heyningen**

United Kingdom

**Lut Van Laer**

Belgium

**Rick van Minkelen**

Netherlands

**Barbara Vanderhyden**

Canada

**Marie Vanier**

France

**Roshni Vara**

United Kingdom

**Robert Vassallo**

United States of America

**Sebastiaan Vastert**

Netherlands

**Guillaume Velasco**

France

**Sebastiaan Velthuis**

Netherlands

**Charles Venditti**

United States of America

**Camiel Verhamme**

Netherlands

**Francis Veyckemans**

Belgium

**Valeria Viassolo**

Switzerland

**Michel Vidaud**

France

**Catherine Vignal Clermont**

France

**Lluïsa Vilageliu**

Spain

**Bente Vilsen**

Denmark

**Jerry Vockley**

United States of America

**Bettie Voordouw**

Netherlands

**Thomas Wagner**

Germany

**Zachary Wallace**

United States of America

**Maggie Walter**

Germany

**Zhao Wang**

China

**kentaro Watanabe**

Japan

**Stefanie Weber**

Germany

**Corry Weemaes**

Netherlands

**Neal Weinreb**

United States of America

**Gabrielle Wheway**

United Kingdom

**Dagmar Wieczorek**

Germany

**Frits Wijburg**

Netherlands

**Bridget Wilcken**

Australia

**Ole Winther**

Denmark

**Ingegerd Witt Engerström**

Sweden

**Pierre Wolkenstein**

France

**Amy Wong**

Canada

**Durhane Wong-Rieger**

Canada

**Tim Wood**

United States of America

**Liz Worthey**

United States of America

**Lawrence Wrabetz**

United States of America

**Chen-Chi Wu**

Taiwan

**Takashi Yamamura**

Japan

**Peizeng Yang**

China

**Bing Yao**

United States of America

**Jacques Young**

France

**Peter Young**

Germany

**Bernhard Zabel**

Germany

**Giovanna Zambruno**

Italy

**Andreas Zankl**

Australia

**Ginevra Zanni**

Italy

**Cornelia Zeidler**

Germany

**Sebastian Zeissig**

Germany

**Christina Zeitz**

France

**Wenping Zhao**

United States of America

**Ari Zimran**

Israel

**Christos Zouboulis**

Germany

**Massimo Zuin**

Italy

**Christiane Zweier**

Germany

